# Visual impairment in children with a brain tumor: a prospective nationwide multicenter study using standard visual testing and optical coherence tomography (CCISS study)

**DOI:** 10.1186/s12886-019-1225-8

**Published:** 2019-11-09

**Authors:** M. A. Nuijts, M. H. Degeling, I. Stegeman, A. Y. N. Schouten-van Meeteren, S. M. Imhof

**Affiliations:** 1 Department of Ophthalmology, University Medical Center Utrecht, Utrecht University, Room E 03.136, P.O. Box 85500, 3508 GA Utrecht, The Netherlands; 2 Department of Ophthalmology, University Medical Center Utrecht, Utrecht, The Netherlands; 3 Department of Neuro-Oncology, Princess Máxima Center for Pediatric Oncology, Utrecht, The Netherlands

**Keywords:** Brain tumor, Child, Visual function, Visual impairment, Optical coherence tomography

## Abstract

**Background:**

Children with a brain tumor have a high risk of impaired vision. Up to now, visual acuity measurement, visual field testing and orthoptic testing are the most informative diagnostic investigations for the assessment of visual function. Evaluating vision in children can be challenging given the challenges in cooperation, concentration and age-dependent shifts in visual tests. Since visual loss due to a brain tumor can be progressive and irreversible, we must aim to detect visual impairment as early as possible. Several studies have shown that optical coherence tomography facilitates discovery of nerve fiber damage caused by optic nerve glioma. Consequently, early detection of potential ocular damage will effect treatment decisions and will provide timely referral to visual rehabilitation centers.

**Methods/design:**

The CCISS study is a prospective, observational, multicenter cohort study in The Netherlands. Patients aged 0–18 years with a newly diagnosed brain tumor are invited for inclusion in this study. Follow-up visits are planned at 6, 12, 18 and 24 months. Primary endpoints are visual acuity, visual field and optical coherence tomography parameters (retinal nerve fiber layer thickness and ganglion cell layer – inner plexiform layer thickness). Secondary endpoints include the course of visual function (measured by visual acuity, visual field and optical coherence tomography at different follow-up visits), course of the disease and types of treatment.

**Discussion:**

The CCISS study will heighten the awareness of visual impairment in different types of brain tumors in children. This study will show whether optical coherence tomography leads to earlier detection of visual impairment compared to standard ophthalmological testing (i.e. visual acuity, visual field testing) in children with a brain tumor. Furthermore, the systematic approach of ophthalmological follow-up in this study will give us insight in the longitudinal relation between the course of visual function, course of the disease and types of treatment in children with a brain tumor.

**Trial registration:**

The CCISS study is prospectively registered in the Netherlands Trial Register (NTR) since April 2019. Identifier: NL7697.

## Background

Brain tumors are the second most common malignancies in children with an age-standardised incidence of 4 / 100.000 [1, 2]. Due to improvements in diagnostics and treatment, the overall survival rate of children with a brain tumor has improved [3, 4]. However, survivors of a brain tumor are at risk for severe late effects of the disease and its treatment [5]. Visual impairment is one of these severe late sequelae of a pediatric brain tumor [6, 7]. It is reported that the harmful influence of the disease and / or its treatment on visual functioning has great impact on the general psychomotor development, school participation and societal participation later in life [7, 8].

Brain tumors can cause visual impairment by affecting both the afferent and efferent visual pathway. Important predictors for visual impairment are tumor location and elevated intracranial pressure (ICP) [9, 10]. Compression of the visual pathway by the tumor may lead to decreased visual acuity (VA), visual field (VF) loss and ocular motility deficits [6, 7, 11]. Furthermore, obstructive hydrocephalus, mass effect of the brain tumor, brain edema and leptomeningeal involvement by the tumor can cause elevated ICP. Elevated ICP can eventually lead to papilledema and optic disc atrophy, causing visual loss as well [12]. In addition, visual impairment can also be the consequence of different therapies such as surgery, radiation and chemotherapy [13]. Surgical resection of the brain tumor can lead to visual impairment via direct surgical trauma of the optic pathway or via perioperative visual loss. Perioperative visual loss can be the result of abrupt decrease in ICP or interruption of the vascular supply of visual structures [14, 15]. Furthermore, radiation can lead to radiation-induced optic neuropathy and/or radiation necrosis of the visual system [16–18]. Finally, different types of chemotherapy can cause optic neuritis, optic neuropathy, papilledema, maculopathy and cataracts [19, 20]. Early detection of visual impairment is crucial because visual loss due to a brain tumor or its treatment is often irreversible [21]. In addition, the selection of the most accurate diagnostic testing methods for monitoring visual function per age and type of brain tumor is still inconsistent. Currently, each center uses its own schedule for checkups since there is no international standardization of vision testing for this patient group. Standardization of diagnostic testing methods will optimize earlier detection of changes in visual function, initiation of early treatment to preserve visual function and provide timely referral to visual rehabilitation centers to improve coping with aspects of development in daily life [22].

Testing of VA is most commonly used as an ophthalmological endpoint in children with a brain tumor. Other frequently used ophthalmological testing methods include VF examination, funduscopy, color and contrast vision and neurophysiological assessment with visual evoked potentials. However, these tests are often not possible in (young) children because of limitations in cooperation and communication or the tests are too burdensome to perform repeatedly [7, 23–25]. Several studies have shown promising results for retinal optical coherence tomography (OCT) as an objective and consistent method for ophthalmological follow up in children with optic pathway glioma or craniopharyngioma [26–30]. Optical coherence tomography makes use of infrared light waves to measure the thickness of separate retinal layers [31, 32]. The relation between an abnormal retinal nerve fiber layer (RNFL) thickness and macular ganglion cell layer – inner plexiform layer (GCL-IPL) thickness on one side and decline in VA and VF for children with optic pathway glioma (OPG) on the other side has already been established [29, 33, 34]. However, studies evaluating the relevance of VA testing, VF testing and OCT for monitoring visual function in different subgroups of childhood brain tumors at different time points of follow-up are not available yet.

The results of our CCISS study: “Child Central nervous tumors InSight in Sight” will provide information on the development of the visual function in children with different types of brain tumors in the Netherlands. The aim of this study is to investigate whether OCT leads to earlier detection of visual impairment compared to standard ophthalmological testing (VA, VF) in children with a brain tumor. Furthermore, this study will provide information about the longitudinal relation between the course of visual function, course of the disease and types of treatment in children with a brain tumor.

## Methods/design

### Study objectives

The primary objective of the study is to investigate whether changes in OCT parameters (thickness of RNFL and GCL-IPL) lead to earlier detection of visual impairment compared to standard ophthalmological testing (VA, VF testing) in children with a brain tumor. Secondary objectives in this study focus on the longitudinal relation between the course of visual function, course of disease and types of treatment in children with a brain tumor.

### Study design and setting

We will perform a quantitative prospective, observational, national cohort study in the Netherlands. This multicenter study is embedded in the University Medical Center Utrecht and will be carried out at Amsterdam University Medical Center, Erasmus Medical Center Rotterdam, Princess Máxima Center for Pediatric Oncology Utrecht, University Medical Center Groningen and University Medical Center Utrecht. The Princess Máxima Center for Pediatric Oncology Utrecht is recently opened as main pediatric oncology center in The Netherlands and therefore the largest cohort of patients will be included here. Selection, invitation and inclusion of patients and performing of ophthalmological tests can take place in all of the abovementioned study sites. Outcomes will be measured at baseline and after six, 12, 18 and 24 months from baseline. Baseline measurement will take place within 4 weeks after the patient is diagnosed with a brain tumor. Allowance of variation around follow-up measurements will be 4 weeks. Depending on tumor type and ophthalmological findings, additional ophthalmological measurements may be necessary and will be performed in the context of patient care.

### Study population

Children, aged 0–18 years old, who are newly diagnosed with a brain tumor in the Netherlands between May 15th 2019 and May 15th 2021 are eligible for participation in the study.

### Recruitment and informed consent procedure

The pediatric oncologist, neurosurgeon or ophthalmologist from the cooperating centers will select patients for invitation. Oral and written explanation will be provided about the purpose of the study and information on any risks and potential discomfort that could be experienced during the study. Written informed consent will be obtained from the parents or guardians of each patient and of the patients older than 12 years of age themselves. Study withdrawal is possible at any moment without providing the reason.

### Study procedures

#### Standard ophthalmological tests

Visual function of the patients will be examined by age-adapted standard ophthalmological tests and OCT. Standard ophthalmological assessment includes orthoptic evaluation, VA, fundus examination and VF testing. We schedule a total of four follow-up visits at 6, 12, 18, and 24 months after inclusion (see Table 1).

**Table 1:** Examination schedule

Assessment	Baseline	6 months	12 months	18 months	24 months
Check eligibility	x				
Informed consent	x				
Demographic data	x				
Check treatment plan	x	x	x	x	x
MRI	x	x	x	x	x
Ophthalmological assessment					
• Orthoptic examination	x	x	x	x	x
• Visual acuity	x	x	x	x	x
• Refraction	(x)^a^	(x)^a^	(x)^a^	(x)^a^	(x)^a^
• Slit lamp	(x)^a^	(x)^a^	(x)^a^	(x)^a^	(x)^a^
• Funduscopy	x	x	x	x	x
Visual fields	x	x	x	x	x
Optical Coherence Tomography	x	x	x	x	x
Adverse events	x	x	x	x	x

^a^ If there is a clinical indication

Orthoptic examination includes inspection/observation of the eyes, eye position tests, ocular motility and convergence, relative afferent pupillary defect (RAPD), color vision test, stereopsis and VA.

Visual acuity testing will be performed for each eye separately (monocular VA). Binocular VA testing will be performed if monocular VA testing fails, for example if the patient is not able to focus and concentrate consistently during testing. Type of VA test will be chosen based on the child’s age, cognitive level and ability to cooperate [23, 35]. Teller acuity cards (TAC) will be used for infants and preverbal toddlers. If possible, Kay Pictures, E-charts, Snellen or numeral charts will be chosen for patients aged 2–3 years old, 3–5 years old and 6 years old or older, respectively.

Funduscopy will be performed to assess the optic disc for the presence of swelling. Presence of papilledema will be considered if there is disc elevation, retinal vessel obscuration or blurred disc margin. The optic disc is graded according to the modified Frisén Scale. The modified Frisén Scale characterizes disc swelling as Grades 0–5, indicating increasing severity of optic disc edema with Grade 0: normal optic disc, and Grade 5: severe degree of edema [36]. Optic nerve head pallor will also be noted. Visual field will be tested according to age-appropriate methods using either the Behavioral Visual Field (BEFIE) Screening test [37], the Humphrey Visual Field Analyzer (HFA) (SITA 24–2 FAST algorithm) [38], the semiautomatic-static Peritest [39] or Goldmann kinetic perimetry [40]. Patient age and cooperation and availability of perimetry instruments per study site determines VF testing method. Most likely a BEFIE test will be performed for children aged 0–5 years. Children old enough (between 5 and 6 years) – 18 years will complete a HFA 24–2 SITA-FAST test, a non-automated static peritest or Goldmann perimetry [37, 41, 42]. Quality of each perimetry test will be assessed using the Examiner Based Assessment of Reliability (EBAR) [42].

#### Optical coherence tomography

Measurements of circumpapillary RNFL thickness and macular GCL-ILP thickness will be performed in all participants using OCT. Children old enough (aged 5–18 years old) and with ability to cooperate (sitting upright and being able to focus for at least 5 min) will be examined with a table-top spectral domain OCT scan (SD Cirrus OCT; Carl Zeiss Meditec, Dublin, CA) using Optic Disc Cube 200 × 200 cube and Macular Cube 200 × 200 protocols (software version 7.0.1.290). For all young patients (between 0 and 5 years old) a handheld OCT-scan (Bioptigen, Research Triangle Park, North Carolina, USA) will be performed under general anesthesia directly after a scheduled MRI-scan [43]. Thirty minutes before undergoing MRI, all patients will receive mydriatic eye drops (0.5% tropicamide and 2.5% phenylephrine). Once optimal image quality is achieved with handheld OCT scanning, a 12 × 12-mm image will be acquired with 600 A-scans per 80 B-scans. Working distance between the handheld OCT probe and the cornea will be based on the child’s axial length and adjusted according to the Pediatric Calculation Table (Bioptigen) adapted from previous recommendations [44].

An overview of age based ophthalmological assessments for study purpose is given in Table 2.

**Table 2:** Overview of age based ophthalmological assessments for study purpose

Primary outcome measures	Ophthalmological testing methods			
	0.5–2 years	2–3 years	3–5 years	≥ 6 years
Visual acuity	TAC	KP	E- charts	Numeral/Snellen charts
Visual fields	BEFIE	BEFIE	BEFIE	Peritest, HFA 24–2 SITA-FAST, Goldmann Perimetry
Thickness of retinal layers (RNFL, GCL-IPL)	Handheld OCT	Handheld OCT	Handheld OCT	Table-top OCT

*BEFIE* Behavioral Visual Field Screening test, *GCL-IPL* ganglion cell layer – inner plexiform layer, *HFA* Humphrey Visual Field Analyzer, *KP* Kay Pictures, *OCT* Optical Coherence Tomography, *RNFL* retinal nerve fiber layer, *TAC* Teller Acuity Cards

### Outcomes

#### Baseline characteristics

Baseline variables include age, gender, medical and ophthalmological history, neurofibromatosis 1 (yes/no), brain tumor type (histology if available), MRI tumor location, tumor size and presence/absence of hydrocephalus and/or metastases.

#### Primary outcome measures

Primary outcome measures are visual acuity, visual field and changes in OCT parameters (RNFL thickness and GCL-ILP thickness).

Visual acuity improves with age, most dramatically in the first 24 months of life, followed by a consistent phase of slower improvement continuing up to 72 months of age and likely beyond. We therefore use age-specific VA norms for the definition of normal vision [35, 45]. Clinically relevant change in VA will be defined as a difference of 0.2 logarithm of the minimal angle of resolution (logMAR) VA or more compared to the VA at baseline visit for each eye [29].

Visual field size/area and sensitivity of normal VF increases with age. VF development occurs predominantly in the temporal and inferotemporal field for children between 5 and 12 years [41].

Results of the BEFIE test will be categorized as ‘normal’ when the peripheral visual field (PVF) extended ≥40 degrees nasally and ≥ 70 degrees temporally, corresponding to the maximum measurable VF with the semiautomatic-static Peritest method [37]. Age-dependent pathological limits for PVF are available for patients under five years of age [46]. An abnormal PVF will be subclassified into symmetric (concentric) PVF defects and asymmetric or homonymous PVF defects. Further subclassification of asymmetric or homonymous PFV defects is made in the article of Koenraads [37]. Visual field loss using HFA24–2 SITA-FAST will be defined as three or more contiguous points reaching significance (*P* < 0.05). Humphrey VF tests will be included if false-positive errors, false negative errors and fixation losses will be less than 20%. Goldmann perimetry will be performed using V-4-E and I-4-E isopters. VF loss using Goldmann perimetry will be defined as any constriction greater than 10 degrees across a minimum of 3 contiguous 15 degree vectors [29].

Changes in OCT parameters will be determined by measuring RNFL thickness and GCL-ILP thickness at different time points. To account for differences between patient specific circumpapillary RNFL thickness and macular GCL-IPL thickness values at study entry, OCT devices and OCT segmentation algorithms, change in circumpapillary RNFL thickness and macular GCL-IPL thickness will be calculated as a percent change from baseline. A change in circumpapillary RNFL thickness and/or macular GCL-ILP thickness of ≥10% from baseline will be chosen based on previously published data [29, 34, 47, 48].

#### Secondary outcome measures

Secondary outcome measures include the course of visual function, course of disease and types of treatment. Visual function will be measured by the above-mentioned primary outcome measures in a follow-up period of 2 years. Disease status will be determined by MRI including tumor size, tumor location, hydrocephalus score and presence or absence of metastases. Optic pathway gliomas will be classified using the modified Dodge classification [49]. Treatment characteristics will include the following types of treatment: 1) neurosurgery; 2) systemic therapy (chemotherapy and/ or targeted therapy; and 3) radiotherapy. Neurosurgical procedures will be defined by biopsy, tumor resection (partial, near total or total) and neurosurgical procedures in patients presenting with hydrocephalus (e.g. external ventricular drain (EVD), endoscopic third ventriculostomy (ETV) and/or ventriculperitoneal (VP) shunt). Systemic therapy will be defined by type and dose of drug and duration of treatment. Radiotherapy will be defined by local/craniospinal and total dose in Gray (Gy).

#### Adverse events

Adverse events are defined as any undesirable experience occurring to a subject during the study, whether or not considered related to the ophthalmological tests. All adverse events reported spontaneously by the subject or observed by the investigator or his staff will be recorded if they occur within 24 h after the ophthalmological examination.

### Statistical analysis

All baseline variables will be summarized as the distribution of frequency, absolute and percentage. For all baseline variables, synthesis tables containing mean, standard deviation, maximum and minimum will be produced. Data on the outcomes of VA testing, VF and OCT are continues variables. The visual function at diagnosis of a childhood brain tumor measured with VA and VF testing will be reported for tumor subgroups. Data which is missing completely at random (MCAR), will be handled with multiple imputation. With 10 imputations, pooled estimates will be reported. As a sensitivity analysis, a complete case analyses will be presented.

The predictive value of OCT will be investigated by estimating two logistic regression model, separate for VA and VF. The outcome variables are the presence/absence of VA loss and VF loss. Since OCT will be performed at different time points, a mixed model will be carried out to study the association between OCT and the outcome, taking the repeated measurement design of the study into account.

The longitudinal relation between the course of visual function, course of disease and types of treatment will be assessed with linear mixed models. Confounding factors could possibly be medical and ophthalmological history, presence of neurofibromatosis, type of tumor, tumor size, tumor location, metastasis and/or the presence of hydrocephalus.

Statistical analysis will be performed with the Statistical Package for the Social Sciences (SPSS, Chicago, IL, USA), version 25.0.0.2.

### Sample size

The incidence of a childhood brain tumor in the Netherlands is about 120 per year [50]. This observational study aims to include a nationwide cohort children diagnosed with a brain tumor between May 15th 2019 and May 15th 2021. We estimate a participation rate of 70% since no age-limits and / or barriers like extra burden for the child and / or language difficulties are expected (*N* = 168). We expect 70% of those children to be at risk of developing visual impairment. Thirty percent of those 168 (*N* = 50) children do not have a risk of developing visual impairment. In logistic regression analysis, 1 variable per 10 patients with the outcome can be included, therefore we expect to be able to correct for 4 confounding factors.

### Data management

Personal data will be handled confidentially and according to Good Clinical Practice (GCP) guidelines. The handling of personal data will comply with the General Data Protection Regulation. Patient data will be collected in the certified electronic data capture tool “Castor”. Personal patient information (e.g. name and date of birth) will be stored in the participating center separately from the research data. A subject identification code list will be used to link the research data to personal patient information. The subject identification code list will be safeguarded by the coordinating researcher per participating center. All data and documents will be archived by the members of the research team for 15 years.

## Discussion

Numerous visual problems have been reported in children with a brain tumor. However, the most appropriate ophthalmological testing methods for monitoring the visual function in this patient group are still unknown. Accurate testing of visual function and timely detection of visual problems will lead to improved patient care and improved quality of life for children diagnosed with a brain tumor. The primary aim of the CCISS study is to assess whether OCT leads to earlier detection of visual decline compared to VA and VF testing in children with a brain tumor. Optical coherence tomography can also be successfully performed in young and non-cooperative children by using a handheld OCT equipment, whereas VA and VF testing are less reliable since they require patient’s collaboration. More reliable outcome data for vision can be supportive to improve upcoming treatment decisions. Furthermore, we will collect high quality data on the longitudinal relation between the course of visual function, disease status and type of treatment in children with a brain tumor at standardized follow-up moments.

## Study status

The final protocol version is 2.0 and date April 2019. This study is currently ongoing. Recruitment of patients has started on 15 May 2019 and we expect the recruitment to be complete by 15 May 2021.

## Data Availability

Data sharing is not applicable to this article as no datasets were generated or analysed in this study protocol.

## Abbreviations

BEFIE test: Behavioral Visual Field Screening test
CCISS: Child Central nervous tumors InSight in Sight
EBAR: Examiner Based Assessment of Reliability
ETV: Endoscopic third ventriculostomy
EVD: External ventricular drain
GCL-IPL: Ganglion cell layer – inner plexiform layer
GCP: Good Clinical Practice
Gy: Gray
HFA: Humphrey Visual Field Analyzer
ICP: Intracranial pressure
KP: Kay Pictures
logMAR: Logarithm of the minimal angle of resolution
MCAR: Missing completely at random
NTR: Netherlans Trial Register
OCT: Optical Coherence Tomography
PVF: Peripheral visual field
RAPD: Relative afferent pupillary defect
RNFL: Retinal nerve fiber layer
TAC: Teller Acuity Cards
VA: Visual acuity
VF: Visual field
VP shunt: Ventriculperitoneal shunt
